# Changes in Workers’ Sedentary and Physical Activity Behaviors in Response to the COVID-19 Pandemic and Their Relationships With Fatigue: Longitudinal Online Study

**DOI:** 10.2196/26293

**Published:** 2021-03-26

**Authors:** Mohammad Javad Koohsari, Tomoki Nakaya, Gavin R McCormack, Ai Shibata, Kaori Ishii, Koichiro Oka

**Affiliations:** 1 Faculty of Sport Sciences Waseda University Tokorozawa Japan; 2 Melbourne School of Population and Global Health The University of Melbourne Melbourne Australia; 3 Behavioural Epidemiology Laboratory Baker Heart and Diabetes Institute Melbourne Australia; 4 Graduate School of Environmental Studies Tohoku University Sendai Japan; 5 Department of Community Health Sciences Cumming School of Medicine University of Calgary Calgary, AB Canada; 6 Faculty of Kinesiology University of Calgary Calgary, AB Canada; 7 School of Architecture, Planning and Landscape University of Calgary Calgary, AB Canada; 8 Faculty of Health and Sport Sciences University of Tsukuba Tsukuba Japan

**Keywords:** COVID-19, physical inactivity, sitting time, mental health, Japan, prospective design

## Abstract

**Background:**

Sedentary behaviors and physical activity are likely to be affected by the COVID-19 outbreak, and sedentary lifestyles can increase subjective fatigue. The nonpharmaceutical policies imposed as a result of the COVID-19 pandemic may also have adverse effects on fatigue.

**Objective:**

This study has two aims: to examine the changes in sedentary behaviors and physical activity of company workers in response to the COVID-19 pandemic in Japan and to examine relationships between changes in these sedentary behaviors and physical activity and changes in fatigue.

**Methods:**

Data from a nationwide prospective online survey conducted in 2019 and 2020 were used. On February 22, 2019, an email with a link to participate in the study was sent to 45,659 workers, aged 20 to 59 years, who were randomly selected from a database of approximately 1 million individuals. A total of 2466 and 1318 participants, who self-reported their occupation as company workers, answered the baseline and follow-up surveys, respectively. Surveys captured fatigue, workday and daily domain-specific sedentary behaviors and physical activity, and total sedentary behaviors and physical activity. We used multivariable linear regression models to estimate associations of changes in sedentary behaviors and physical activity with changes in fatigue.

**Results:**

Increases in public transportation sitting during workdays, other leisure sitting time during workdays, and other leisure sitting time were associated with an increase in the motivation aspect of fatigue (*b*=0.29, 95% CI 0-0.57, *P*=.048; *b*=0.40, 95% CI 0.18-0.62, *P*<.001; and *b*=0.26, 95% CI 0.07-0.45, *P*=.007, respectively). Increases in work-related sitting time during workdays, total sitting time during workdays, and total work-related sitting time were significantly associated with an increase in the physical activity aspect of fatigue (*b*=0.06, 95% CI 0-0.12, *P*=.03; *b*=0.05, 95% CI 0.01-0.09, *P*=.02; and *b*=0.07, 95% CI 0-0.14, *P*=.04, respectively). The motivation and physical activity aspects of fatigue increased by 0.06 for each 1-hour increase in total sitting time between baseline and follow-up (*b*=0.06, 95% CI 0-0.11, *P*=.045; and *b*=0.06, 95% CI 0.01-0.10, *P*=.009, respectively).

**Conclusions:**

Our findings demonstrated that sedentary and active behaviors among company workers in Japan were negatively affected during the COVID-19 outbreak. Increases in several domain-specific sedentary behaviors also contributed to unfavorable changes in workers’ fatigue. Social distancing and teleworking amid a pandemic may contribute to the sedentary lifestyle of company workers. Public health interventions are needed to mitigate the negative effects of the COVID-19 pandemic or future pandemics on sedentary and physical activity behaviors and fatigue among company workers.

## Introduction

Sedentary lifestyles can increase the risk of many chronic diseases [1] and can increase subjective fatigue in working adults [2,3]. Subjective fatigue refers to “an overwhelming sense of tiredness, lack of energy, or feeling of exhaustion” [4]. Subjective fatigue has several adverse effects on health and well-being, productivity, and safety among workers [5-7]. For example, a population-based study conducted in Germany found that chronic fatigue was negatively associated with quality of life [8].

While the exact causes of fatigue in workplaces have not yet been fully investigated [9], promoting physical activity and interrupting sedentary time may mitigate subjective fatigue at work [10]. For example, a randomized trial study found that breaking prolonged sitting time with walking breaks was associated with lower fatigue levels among a sample of overweight and obese adults [11]. Longer sitting time was also found to be associated with more fatigue in a sample of Swedish adults [12]. Nevertheless, this evidence is mainly based on cross-sectional studies that were conducted among high-risk groups, such as overweight and obese individuals. Further research is warranted to assess whether longitudinal changes in sedentary and active behaviors may influence general workers’ fatigue.

The World Health Organization declared COVID-19 a pandemic on March 11, 2020 [13]. Since then, several social distancing policies, such as school and university closures, recommendations for working from home, and bans on large gatherings, have been implemented by governments and local authorities to slow the spread of COVID-19. In Japan, a state of emergency was declared on April 7, 2020, by the government to contain the spread of COVID-19 [14]. Under this state of emergency, prefectural governors were allowed to request that residents restrict their unnecessary trips and stay at home as much as possible; they also asked that public places, such as cinemas and museums, limit their operations. However, in contrast with several other countries, no penalty or legal force under Japanese law existed for those people or companies who disobeyed the lockdown request. Nevertheless, sedentary behaviors and physical activity are likely to be affected by these social distancing policies. A worldwide study of the effects of COVID-19 on physical activity found a rapid decrease in step counts, an indicator of physical activity, across multiple countries [15]. The nonpharmaceutical policies resulting from public health measures in place to reduce person-to-person transmission of COVID-19 may also have adverse effects on people’s fatigue. For instance, a study conducted in Poland reported a high level of everyday fatigue (ie, measured through items capturing physical, mental, and social fatigue) during the COVID-19 home quarantine period compared to before the quarantine period [16]. Since it is currently unknown how long the COVID-19 pandemic will last, workers may need to follow workplace social distancing policies for an unpredictable duration. Even after this pandemic ends, it is likely that workers will continue to be sedentary due to advancements in workplace technology. Given the links between fatigue and an active lifestyle, further research is needed to shed light on how COVID-19 has influenced the sedentary behaviors and physical activities of different population groups, especially company workers who are already exposed to a sedentary lifestyle.

To our knowledge, no study has examined longitudinal changes in sedentary behaviors and physical activity among company workers during the COVID-19 pandemic and their effects on fatigue. Therefore, this study has two aims: (1) examine the changes in sedentary behaviors and physical activity of company workers during the COVID-19 outbreak in Japan and (2) examine the relationships between these changes in sedentary behaviors and physical activity and fatigue.

## Methods

### Data Source and Participants

Our study included data from a nationwide prospective online survey conducted in 2019 and 2020. The participants were recruited from a group of registered individuals of a Japanese internet research service company (MyVoice Communication, Inc). Approximately 1 million individuals across Japan voluntarily participated in populating this company’s database and registered their sociodemographic information. The eligibility criteria for study participation were based on having the same number of participants of each sex (ie, female and male) and each age group (ie, workers in their 20s, 30s, 40s, and 50s) and being a company worker. Given our research budget, we aimed to recruit a total of 3200 workers, aged 20 to 59 years: 1600 participants from each sex category and 800 participants from each age group. On February 22, 2019, an email with a link to participate in the study was sent to 45,659 individuals who were randomly selected from the database by sex and age strata; the company sent emails until the planned sample size was reached. Of these, 2921 individuals were eligible to participate in the follow-up study on July 8, 2020—279 respondents had withdrawn from the company’s database—and 1709 completed the follow-up survey (1709/3200, 53.4% of the baseline participants). Only participants who self-reported their occupation as company workers were included in this study: 2466 and 1318 participants at baseline and follow-up, respectively. The follow-up time frame had not been originally intended, and it was in response to the COVID-19 pandemic. Cash rewards valued at ¥140 (US $1.30) and ¥120 (US $1.10) were offered as incentives to participate in the first and second surveys, respectively. All participants signed an online informed consent form. The internet research service company removed participant names, addresses, personal health numbers, contact information, and any other details from the data set that might be used to identify individuals prior to transferring the data to researchers. The Institutional Ethics Committee of Waseda University approved this study (2020-135).

### Measures

#### Fatigue

The Japanese version of the Checklist Individual Strength questionnaire (CIS20-R) was used to assess perceived fatigue during the past 2 weeks [17,18]. The CIS20-R is a multidimensional 20-item questionnaire that measures four aspects of fatigue—subjective fatigue, concentration, motivation, and physical activity—as well as total fatigue [17]. This questionnaire has high internal reliability (Cronbach α=.90 for total fatigue and ranges from .83 to .92 for its subscales) [17], and the questionnaire is valid in the working population [18,19]. Participants scored each item on a 7-point Likert scale ranging from 1 (exactly) to 7 (not at all). The scoring protocol for the CIS20-R has been described in detail elsewhere [19]. In brief, higher scores indicate a higher degree of fatigue, more concentration problems, reduced motivation, and less physical activity. Total fatigue was calculated from the sum of the four aspects of fatigue. We estimated absolute changes in total fatigue (ie, total CIS20-R score) and aspects of fatigue before and during the COVID-19 pandemic by subtracting baseline values from follow-up values.

#### Sedentary Behaviors and Physical Activity

Domain-specific sedentary behaviors were assessed using a Japanese, 6-item, self-reported questionnaire with a 1-week recall period [20]. Participants were asked to report their daily average sedentary behaviors over the past 7 days separately for workdays—or weekdays for unemployed individuals—and nonworkdays (ie, weekends) across the following six domains: driving or riding by car; using public transport; at work; watching television, videos, and DVDs; using a computer, cell phone, or tablet PC outside of working hours; and during leisure time, excluding watching television, videos, and DVDs. Participants reported the average time (hours and minutes) spent in each of these categories per day. The reliability and validity of this questionnaire have been reported elsewhere [20]. Average daily values of total sedentary time and each domain’s sedentary time were calculated with weighting for the number of workdays and nonworkdays. The average workday value of total sedentary time was also calculated by summing all six domains for workdays. Domain-specific physical activity was measured using the Global Physical Activity Questionnaire (GPAQ) [21]. The method of cleaning and scoring GPAQ data has been described in detail elsewhere [22]. Briefly, this questionnaire consists of 16 questions that assess self-reported domain-specific physical activity—during work, transport, and leisure—and sitting time in a typical week. It has acceptable reliability and validity in Japanese adults [23]. Average daily hours of work-related vigorous physical activity, work-related moderate physical activity, transport-related physical activity, leisure-related vigorous physical activity, leisure-related moderate physical activity, and total physical activity were calculated. We estimated absolute changes in sedentary behaviors and physical activity before and during the COVID-19 pandemic by subtracting baseline values from follow-up values.

#### Sociodemographic Variables

Participants self-reported their baseline age, sex (ie, female or male), marital status (ie, single or couple), highest education (ie, tertiary, below tertiary, or other), and gross annual individual income (ie, <¥4,000,000 [US $37,000] or ≥¥4,000,000 [US $37,000]).

### Statistical Analysis

Descriptive statistics, including means and SDs as well as frequencies, were calculated for the baseline sociodemographic variables. Paired *t* tests were used to compare the domain-specific sedentary and physical activity behaviors with total fatigue and aspects of fatigue before and during the COVID-19 outbreak. A separate multivariable linear regression model estimated the associations between changes in sedentary behaviors and physical activities and the changes in each fatigue aspect at the individual level. The regression models were adjusted for the sociodemographic variables (ie, age, sex, marital status, highest education, and gross annual household income). The regression models were also adjusted for baseline fatigue to account for the potential effect of baseline values on change scores. Normality assumptions were checked by the quantile-quantile plots of the residuals. For all point estimates, where *b* is the unstandardized regression coefficient, 95% CIs were estimated. A complete-case analysis was chosen because the proportion of missing data for our variables of interest was low (5%) [24]. We found no significant differences in any measured characteristics between those individuals with missing data and those with complete data. Our analysis excluded those who were unable to engage in physical activity due to health issues. Analyses were conducted using Stata 15.0 (Stata Corp), and the level of significance was set at *P*<.05.

## Results

### Sample Characteristics

Table 1 shows the baseline sample characteristics (N=2466). The participants had an average age of 39.6 years (SD 10.7), and the majority were male, were single, had a high tertiary education, and had an annual gross household income lower than ¥4,000,000 (US $37,000). The majority of the participants had sedentary occupations (ie, office-based workers) (1807/2466, 73.3%) and 26.7% (659/2466) had active occupations (eg, manual labor). There were no participants who became unemployed by the time of the follow-up survey. The participants reported the total number of weekly days they worked from home before and during the COVID-19 pandemic. The average number days that the participants worked from home was 0.2 and 1 day per week before and during the COVID-19 pandemic, respectively.

**Table 1:** Baseline characteristics of study participants in Japan, February 2019.

Characteristic		Value (N=2466)
Age in years, mean (SD)		39.6 (10.7)
**Sex, n (%)**		
	Female	1212 (49.1)
	Male	1254 (50.9)
**Marital status, n (%)**		
	Single	1392 (56.4)
	Couple	1074 (43.6)
**Highest education, n (%)**		
	Tertiary	1989 (80.7)
	Below tertiary	477 (19.3)
**Gross annual individual income (¥), n (%)**		
	<4,000,000^a^	1381 (56.0)
	≥4,000,000^a^	1085 (44.0)

^a^¥4,000,000 is equivalent to US $37,000.

### Changes in Sedentary Behaviors, Physical Activity, and Fatigue

Mean hours per day of sedentary behaviors and physical activity before and during the COVID-19 pandemic are shown in Tables 2 and 3. These comparisons were restricted only to participants who answered both surveys. There were several significant differences between workers’ domain-specific sedentary behaviors from one period to the next: participants spent more sedentary time in *work-related sitting time during workday*, *TV viewing time during workday*, *PC use sitting time during workday*, *total sitting time during workday*, *work-related sitting time*, *PC use sitting time*, and *total sitting time* during the COVID-19 pandemic (see Table 2). Participants also reported significantly lower amounts of several domain-specific physical activities during the COVID-19 outbreak: the mean values of vigorous leisure physical activity and total physical activity were significantly lower during the COVID-19 outbreak (see Table 3).

At baseline, the Cronbach α for internal consistency was .87, .75, .56, and .28 for the subjective fatigue, concentration, motivation, and physical activity items, respectively. These estimates were .86, .78, .59, and .33 for the follow-up items, respectively. Despite a small difference in mean values between subjective fatigue before and during the COVID-19 outbreak, this difference was nevertheless statistically significant: higher scores refer to a higher degree of fatigue. No significant difference was observed for the means of the three fatigue aspects of concentration, motivation, and physical activity nor for total fatigue (ie, total CIS20-R score) before and during the COVID-19 pandemic (see Table 4).

Higher scores refer to a higher degree of subjective fatigue, more concentration problems, reduced motivation, less physical activity, and higher total fatigue (ie, total CIS20-R score).

**Table 2:** Workers’ domain-specific sedentary behaviors before (February 2019) and during (July 2020) the COVID-19 outbreak in Japan (n=1086).

Sedentary behavior	Hours per day, mean (SD)			*P* value^a^		Difference, mean (SD)
	Before	During				
Car sitting time during workday	0.45 (0.86)	0.45 (0.87)	.80		0.01 (0.85)	
Public transportation sitting during workday	0.46 (0.72)	0.49 (0.79)	.15		0.03 (0.72)	
Work-related sitting time during workday	5.18 (3.26)	5.69 (3.30)	<.001		0.50 (2.93)	
TV viewing time during workday	1.56 (1.36)	1.65 (1.36)	.03		0.09 (1.27)	
PC use sitting time during workday	1.36 (1.27)	1.47 (1.27)	.01		0.11 (1.40)	
Other leisure sitting time during workday	0.52 (0.69)	0.54 (0.73)	.53		0.02 (0.92)	
Total sitting time during workday	9.53 (3.96)	10.29 (3.99)	<.001		0.75 (3.95)	
Car sitting time	0.50 (0.78)	0.50 (0.76)	.90		0 (0.72)	
Public transportation sitting	0.39 (0.58)	0.39 (0.61)	.80		0 (0.57)	
Work-related sitting time	3.91 (2.49)	4.22 (2.52)	<.001		0.31 (2.32)	
TV viewing time	1.89 (1.54)	1.97 (1.56)	.06		0.08 (1.45)	
PC use sitting time	1.61 (1.41)	1.76 (1.45)	<.001		0.14 (1.47)	
Other leisure sitting time	0.67 (0.83)	0.69 (0.88)	.48		0.02 (1.09)	
Total sitting time	8.96 (3.53)	9.53 (3.59)	<.001		0.56 (3.67)	

^a^*P* values were based on paired-sample *t* tests.

**Table 3:** Workers’ domain-specific physical activity before (February 2019) and during (July 2020) the COVID-19 outbreak in Japan (n=1315).

Physical activity	Hours per day, mean (SD)		*P* value^a^	Difference, mean (SD)
	Before	During		
Work-related vigorous physical activity	0.21 (1.03)	0.19 (0.84)	.40	–0.02 (1.06)
Work-related moderate physical activity	0.43 (1.32)	0.39 (1.17)	.24	–0.05 (1.43)
Transport-related physical activity	0.48 (0.72)	0.44 (0.73)	.10	–0.04 (0.83)
Leisure-related vigorous physical activity	0.25 (0.72)	0.20 (0.53)	.01	–0.05 (0.64)
Leisure-related moderate physical activity	0.37 (0.83)	0.33 (0.72)	.07	–0.04 (0.80)
Total physical activity	1.75 (2.79)	1.55 (2.31)	.01	–0.20 (2.61)

^a^*P* values were based on paired-sample *t* tests.

**Table 4:** Workers’ total fatigue and aspects of fatigue before (February 2019) and during (July 2020) the COVID-19 outbreak in Japan (n=1318).

Fatigue aspect	CIS20-R^a^ score, mean (SD)		*P* value^b^	
	Before	During		
Subjective fatigue	31.2 (9.4)	31.9 (9.1)	.002	
Concentration	20.1 (4.4)	20.3 (4.4)	.24	
Motivation	17.4 (4.2)	17.3 (4.2)	.33	
Physical activity	12.3 (3.0)	12.2 (3.0)	.45	
Total fatigue	81.0 (16.9)	81.6 (16.6)	.10	

^a^CIS20-R: Checklist Individual Strength questionnaire.

^b^*P* values were based on paired-sample *t* tests.

### Associations of Changes in Sedentary Behaviors and Physical Activity With Fatigue

Table 5 shows the associations of the changes in workers’ total fatigue (ie, total CIS20-R score) and aspects of fatigue with those in workers’ domain-specific sedentary behaviors. Increases in public transportation sitting during workdays, other leisure sitting times during workdays, and other leisure sitting times were significantly associated with an increase in the motivation aspect of fatigue. Increases in work-related sitting time during workdays, total sitting time during workdays, and work-related sitting time were significantly associated with an increase in the physical activity aspect of fatigue. The subscale scores for the motivation and physical activity aspects of fatigue increased by 0.03 for each half-hour increase in total sitting time between baseline and follow-up (*b/2*=0.03, 95% CI 0-0.05 and 95% CI 0.01-0.05, respectively). None of the changes in domain-specific physical activity were significantly associated with workers’ total fatigue (ie, total CIS20-R score) or fatigue subscale scores (see Table 6).

**Table 5:** Results of multivariable linear regression models assessing associations between absolute changes in workers’ domain-specific sedentary behaviors and changes in workers’ total fatigue and aspects of fatigue in Japan (n=1086).

Sedentary behavior	Subjective fatigue, *b*^a^ (95% CI)	Concentration, *b* (95% CI)	Motivation, *b* (95% CI)	Physical activity, *b* (95% CI)	Total fatigue, *b* (95% CI)
Car sitting time during workday	–0.05 (–0.55 to 0.45)	–0.04 (–0.30 to 0.22)	–0.01 (–0.25 to 0.24)	0.03 (–0.16 to 0.22)	–0.05 (–0.94 to 0.85)
Public transportation sitting during workday	–0.23 (–0.82 to 0.36)	–0.08 (–0.38 to 0.23)	*0.29* *(0 to 0.57)* ^*b*^	0.04 (–0.19 to 0.26)	0.01 (–1.04 to 1.06)
Work-related sitting time during workday	0.02 (–0.13 to 0.16)	0.07 (–0.01 to 0.14)	0.02 (–0.05 to 0.10)	*0.06* *(0 to 0.12)*	0.16 (–0.10 to 0.42)
TV viewing time during workday	0.07 (–0.27 to 0.40)	0.06 (–0.11 to 0.23)	–0.02 (–0.18 to 0.14)	0.04 (–0.09 to 0.17)	0.16 (–0.44 to 0.76)
PC use sitting time during workday	0.05 (–0.25 to 0.35)	–0.13 (–0.28 to 0.03)	–0.02 (–0.16 to 0.13)	0.03 (–0.09 to 0.14)	–0.06 (–0.61 to 0.48)
Other leisure sitting time during workday	0.30 (–0.17 to 0.76)	0.02 (–0.22 to 0.26)	*0.40* *(0.18 to 0.62)*	0.12 (–0.06 to 0.29)	0.76 (–0.08 to 1.59)
Total sitting time during workday	0.03 (–0.08 to 0.14)	0.02 (–0.03 to 0.08)	0.04 (–0.01 to 0.09)	*0.05* *(0.01 to 0.09)*	0.14 (–0.06 to 0.33)
Car sitting time	0.23 (–0.36 to 0.82)	0.07 (–0.24 to 0.37)	0.09 (–0.20 to 0.37)	0.10 (–0.13 to 0.32)	0.50 (–0.55 to 1.56)
Public transportation sitting	–0.27 (–1.01 to 0.47)	–0.13 (–0.51 to 0.26)	0.31 (–0.05 to 0.67)	0.05 (–0.23 to 0.33)	–0.02 (–1.34 to 1.31)
Work-related sitting time	–0.02 (–0.21 to 0.16)	0.07 (–0.02 to 0.17)	0.03 (–0.06 to 0.12)	*0.07* *(0 to 0.14)*	0.15 (–0.18 to 0.48)
TV viewing time	0.11 (–0.19 to 0.40)	0.11 (–0.04 to 0.26)	0.03 (–0.11 to 0.17)	0.07 (–0.04 to 0.18)	0.33 (–0.20 to 0.85)
PC use sitting time	–0.02 (–0.31 to 0.27)	–0.11 (–0.26 to 0.04)	0.03 (–0.11 to 0.17)	0.04 (–0.07 to 0.15)	–0.05 (–0.57 to 0.46)
Other leisure sitting time	0.23 (–0.16 to 0.62)	0.02 (–0.18 to 0.22)	*0.26* *(0.07 to 0.45)*	0.09 (–0.06 to 0.24)	0.52 (–0.18 to 1.21)
Total sitting time	0.03 (–0.09 to 0.14)	0.03 (–0.03 to 0.09)	*0.06* *(0 to 0.11)*	*0.06* *(0.01 to 0.10)*	0.17 (–0.04 to 0.36)

^a^*b* is the unstandardized regression coefficient; based on the Checklist Individual Strength questionnaire (CIS20-R) scores. All models were adjusted for age, sex, marital status, highest education, gross annual household income, and baseline fatigue.

^b^Italicized values are statistically significant at *P*<.05.

**Table 6:** Results of multivariable linear regression models assessing associations between absolute changes in workers’ domain-specific physical activity and changes in workers’ total fatigue and aspects of fatigue in Japan (n=1315).

Physical activity	Subjective fatigue, *b*^a^ (95% CI)	Concentration, *b* (95% CI)	Motivation, *b* (95% CI)	Physical activity, *b* (95% CI)	Total fatigue, *b* (95% CI)
Work-related vigorous physical activity	–0.23 (–0.59 to 0.14)	–0.02 (–0.21 to 0.17)	–0.08 (–0.26 to 0.10)	0.01 (–0.13 to 0.15)	–0.30 (–0.95 to 0.35)
Work-related moderate physical activity	–0.10 (–0.37 to 0.17)	–0.07 (–0.21 to 0.07)	–0.03 (–0.16 to 0.10)	0.05 (–0.05 to 0.15)	–0.18 (–0.66 to 0.30)
Transport-related physical activity	–0.06 (–0.52 to 0.40)	–0.02 (–0.26 to 0.22)	0.21 (–0.02 to 0.43)	0.11 (–0.06 to 0.28)	0.20 (–0.63 to 1.02)
Leisure-related vigorous physical activity	–0.26 (–0.86 to 0.33)	–0.17 (–0.48 to 0.15)	0.16 (–0.14 to 0.45)	–0.11 (–0.33 to 0.12)	–0.44 (–1.51 to 0.63)
Leisure-related moderate physical activity	–0.30 (–0.78 to 0.18)	–0.16 (–0.41 to 0.09)	0.13 (–0.11 to 0.37)	–0.04 (–0.22 to 0.14)	–0.30 (–1.16 to 0.57)
Total physical activity	–0.12 (–0.27 to 0.03)	–0.05 (–0.13 to 0.02)	0.02 (–0.05 to 0.09)	0.02 (–0.04 to 0.07)	–0.14 (–0.40 to 0.13)

^a^*b* is the unstandardized regression coefficient; based on the Checklist Individual Strength questionnaire (CIS20-R) scores. All models were adjusted for age, sex, marital status, highest education, gross annual household income, and baseline fatigue.

## Discussion

### Principal Findings

This study is one of the first attempts to empirically examine the longitudinal changes in workers’ domain-specific sedentary behaviors, physical activity, and fatigue during the COVID-19 outbreak. It also investigated the associations of the changes in workers’ sedentary behaviors and physical activity with changes in their fatigue. Compared with before the COVID-19 pandemic, we found that workers spent more time (ie, 5-45 minutes per day) engaged in several domain-specific sedentary behaviors, including work-related sitting, TV viewing time during workdays, PC use sitting time, and total sitting time during the COVID-19 pandemic. They also reported less time engaged in leisure-related vigorous physical activity and total physical activity during the COVID-19 outbreak. Several cross-sectional and longitudinal studies conducted among other population groups echoed similar patterns in how the COVID-19 pandemic unfavorably affected people’s active movement behaviors [25-30]. For example, a national study conducted in Canada found that children and youth reported higher sedentary behaviors and lower physical activity during the COVID-19 outbreak; the study found that less than 5% of children and less than 1% of youth met the combined 24-hour movement behavior goals for physical activity, sedentary behavior, and sleep [25]. Another study conducted in China found that adults’ average steps per day and average moderate- or vigorous-intensity exercise significantly decreased during the semilockdown period of the COVID-19 pandemic [28]. Moreover, a study conducted among hypertensive older adults found that there was an increase in accelerometer-based sedentary behavior and a decrease in physical activity during the COVID-19 pandemic [29]. Our longitudinal findings are the first to confirm that COVID-19-related social distancing policies negatively affect sedentary and active behaviors among company workers, a population already at risk for being physically inactive [31,32].

Our findings also identified changes in sedentary behaviors and physical activity for each domain during the COVID-19 outbreak. Influenced by the government’s self-isolation advice [33], leisure-related vigorous physical activity significantly decreased during the COVID-19 outbreak. The pre-COVID-19 level of leisure-related vigorous physical activity was only 15 minutes per day in our sample, which became 12 minutes per day during the COVID-19 pandemic. Since only a few people engaged in this activity, even in non-COVID-19 times, this pandemic has almost obliterated the vigorous physical activity that took place in the leisure time of the Japanese worker population. We also found that participants reported more work-related sitting time during the COVID-19 outbreak. Company workers usually sit when doing their work, and some of that additional sitting was due to an increase in the use of computers for all work-related meetings during the COVID-19 pandemic. Several companies in Japan have introduced and supported some level of teleworking amid the outbreak of COVID-19. While teleworking may lead to less time traveling for work, it is also likely that teleworking limits the opportunities for employees to accumulate transport-related physical activity and intensifies workers’ sedentary activities, especially during a pandemic. Further studies are needed to investigate the influence of teleworking on workers’ sedentary behaviors and physical activity. We also found that participants spent more sitting time on watching TV or using the PC. One reason for these increases is that people were more likely to avoid going outside following the Japan government’s advice to minimize nonessential trips and reduce train operating hours in some areas. Staying at home limits people’s ability to engage in physical activity and socialize with their friends and family and increases screen sitting time. Future studies can identify home-based interventions to improve people’s active behaviors during a pandemic.

We found that increases in several domain-specific sedentary behaviors were associated with unfavorable changes in the motivation and physical activity aspects of fatigue. Several previous studies have shown adverse effects on fatigue from too much sitting [3,11,12]. While yet to be tested in other populations, public health strategies to reduce sedentary behaviors among company workers could have positive effects on worker fatigue. Reducing sedentary behavior has been highlighted as a more feasible strategy during the COVID-19 pandemic than achieving optimal exercise levels [34]. However, teleworking from home, a new norm resulting from COVID-19-related social distancing policies, may impose a new challenge to reducing occupational sitting time in workers [35]. Future research is needed to identify how teleworking may influence sedentary behaviors and how to develop new policies to reduce workers’ sitting time in the new era of teleworking from home. Furthermore, we found a significant increase in subjective fatigue among workers during the COVID-19 outbreak. Although the exact reasons for increased subjective fatigue in workers during the COVID-19 pandemic remain to be elucidated, the burden of this pandemic on people’s mental health may be involved [36]. Several studies have provided preliminary evidence on the harmful effects of the COVID-19 pandemic on mental health [37-39]. For instance, a national study conducted in the United States found that the prevalence of depression symptoms was more than 3 times higher during the COVID-19 outbreak than before the outbreak [37]. Another study conducted in the Republic of Ireland found that approximately 30% of the participants were screened positive for generalized anxiety disorder or depression during the first week of the COVID-19 lockdown [39]. A sedentary lifestyle, intensified by social distancing policies, may also play a role in the observed increase in worker fatigue since the onset of the COVID-19 pandemic.

### Limitations and Strengths

This study has some limitations. Our self-reported measures of sedentary behaviors and physical activity may be subject to memory and recall bias [40]. While this is a national study of participants recruited from throughout Japan, we are unable to confirm the generalizability of the data from our sample to all company workers. Data on employment status, including full-time or part-time employment, were also unavailable. The physical activity aspect of fatigue had low internal reliability at both baseline and follow-up. Since subjective fatigue may also lead to less physical activity and more sedentary behaviors, the direction of the observed associations remains inconclusive. Our study has several unique strengths, including the use of national data, the focus on the less-studied population group of company workers, and the examination of domain-specific sedentary behaviors and physical activity at different intensities.

### Conclusions

Following the COVID-19 outbreak, there have been many concerns regarding its effects on people’s physical inactivity and chronic diseases [41-43]. Our findings demonstrated that sedentary and active behaviors among company workers in Japan were negatively affected during the COVID-19 outbreak. Increases in several domain-specific sedentary behaviors also contributed to unfavorable changes in workers’ fatigue. Social distancing and teleworking amid a pandemic may contribute to the sedentary lifestyle of company workers. Public health interventions are needed to mitigate the negative effects of the COVID-19 pandemic or future pandemics on sedentary and active behaviors and fatigue among company workers. More evidence is needed to identify the magnitude of changes in sedentary and active behaviors in workers during the COVID-19 outbreak and how these changes may influence workers’ health and well-being.

## Abbreviations

CIS20-R: Checklist Individual Strength questionnaire
GPAQ: Global Physical Activity Questionnaire

## References

[1] Dempsey P, Matthews C, Dashti S, Doherty A, Bergouignan A, van Roekel EH, Dunstan DW, Wareham NJ, Yates TE, Wijndaele K, Lynch BM (2020). Sedentary behavior and chronic disease: Mechanisms and future directions. J Phys Act Health.

[2] de Vries JD, Claessens BJC, van Hooff MLM, Geurts SAE, van den Bossche SNJ, Kompier MAJ (2016). Disentangling longitudinal relations between physical activity, work-related fatigue, and task demands. Int Arch Occup Environ Health.

[3] Thorp AA, Kingwell BA, Owen N, Dunstan DW (2014). Breaking up workplace sitting time with intermittent standing bouts improves fatigue and musculoskeletal discomfort in overweight/obese office workers. Occup Environ Med.

[4] Karlsen K, Larsen JP, Tandberg E, Jørgensen K (1999). Fatigue in patients with Parkinson's disease. Mov Disord.

[5] Saremi M, Fallah MR (2013). Subjective fatigue and medical errors among nurses in an educational hospital. Iran Occup Health.

[6] Sadeghniiat-Haghighi K, Yazdi Z (2015). Fatigue management in the workplace. Ind Psychiatry J.

[7] Connolly D, Fitzpatrick C, O'Toole L, Doran M, O'Shea F (2015). Impact of fatigue in rheumatic diseases in the work environment: A qualitative study. Int J Environ Res Public Health.

[8] Martin A, Chalder T, Rief W, Braehler E (2007). The relationship between chronic fatigue and somatization syndrome: A general population survey. J Psychosom Res.

[9] Williamson A, Friswell R (2013). Fatigue in the workplace: Causes and countermeasures. Fatigue.

[10] Lerman S, Eskin E, Flower D, George E, Gerson B, Hartenbaum N (2012). Fatigue risk management in the workplace. J Occup Environ Med.

[11] Wennberg P, Boraxbekk C, Wheeler M, Howard B, Dempsey PC, Lambert G, Eikelis N, Larsen R, Sethi P, Occleston J, Hernestål-Boman J, Ellis KA, Owen N, Dunstan DW (2016). Acute effects of breaking up prolonged sitting on fatigue and cognition: A pilot study. BMJ Open.

[12] Engberg I, Segerstedt J, Waller G, Wennberg P, Eliasson M (2017). Fatigue in the general population–Associations to age, sex, socioeconomic status, physical activity, sitting time and self-rated health: The northern Sweden MONICA study 2014. BMC Public Health.

[13] Ghebreyesus T (2020). WHO Director-General's opening remarks at the media briefing on COVID-19 - 11 March 2020. World Health Organization.

[14] (2020). [COVID-19] Declaration of a state of emergency in response to the novel coronavirus disease. The Prime Minister of Japan and His Cabinet.

[15] Tison GH, Avram R, Kuhar P, Abreau S, Marcus GM, Pletcher MJ, Olgin JE (2020). Worldwide effect of COVID-19 on physical activity: A descriptive study. Ann Intern Med.

[16] Bartoszek A, Walkowiak D, Bartoszek A, Kardas G (2020). Mental well-being (depression, loneliness, insomnia, daily life fatigue) during COVID-19 related home-confinement-A study from Poland. Int J Environ Res Public Health.

[17] Vercoulen JH, Swanink CM, Fennis JF, Galama JM, van der Meer JW, Bleijenberg G (1994). Dimensional assessment of chronic fatigue syndrome. J Psychosom Res.

[18] Aratake Y, Tanaka K, Wada K, Watanabe M, Katoh N, Sakata Y, Aizawa Y (2007). Development of Japanese version of the Checklist Individual Strength questionnaire in a working population. J Occup Health.

[19] Beurskens A, Bültmann U, Kant I, Vercoulen J, Bleijenberg G, Swaen G (2000). Fatigue among working people: Validity of a questionnaire measure. Occup Environ Med.

[20] Ishii K, Shibata A, Kurita S, Yano S, Inoue S, Sugiyama T, Owen N, Oka K (2018). Validity and reliability of Japanese-language self-reported measures for assessing adults domain-specific sedentary time. J Epidemiol.

[21] Armstrong T, Bull F (2006). Development of the World Health Organization Global Physical Activity Questionnaire (GPAQ). J Public Health.

[22] (2012). Global Physical Activity Questionnaire (GPAQ) Analysis Guide.

[23] Bull F, Maslin T, Armstrong T (2009). Global Physical Activity Questionnaire (GPAQ): Nine country reliability and validity study. J Phys Act Health.

[24] Jakobsen JC, Gluud C, Wetterslev J, Winkel P (2017). When and how should multiple imputation be used for handling missing data in randomised clinical trials - A practical guide with flowcharts. BMC Med Res Methodol.

[25] Moore SA, Faulkner G, Rhodes RE, Brussoni M, Chulak-Bozzer T, Ferguson LJ, Mitra R, O'Reilly N, Spence JC, Vanderloo LM, Tremblay MS (2020). Impact of the COVID-19 virus outbreak on movement and play behaviours of Canadian children and youth: A national survey. Int J Behav Nutr Phys Act.

[26] López-Bueno R, Calatayud J, Andersen LL, Balsalobre-Fernández C, Casaña J, Casajús JA, Smith L, López-Sánchez GF (2020). Immediate impact of the COVID-19 confinement on physical activity levels in Spanish adults. Sustainability.

[27] Wang Y, Zhang Y, Bennell K, White DK, Wei J, Wu Z, He H, Liu S, Luo X, Hu S, Zeng C, Lei G (2020). Physical distancing measures and walking activity in middle-aged and older residents in Changsha, China, during the COVID-19 epidemic period: Longitudinal observational study. J Med Internet Res.

[28] He M, Xian Y, Lv X, He J, Ren Y (2020). Changes in body weight, physical activity, and lifestyle during the semi-lockdown period after the outbreak of COVID-19 in China: An online survey. Disaster Med Public Health Prep.

[29] Browne RA, Macêdo GAD, Cabral LL, Oliveira GT, Vivas A, Fontes EB, Elsangedy HM, Costa EC (2020). Initial impact of the COVID-19 pandemic on physical activity and sedentary behavior in hypertensive older adults: An accelerometer-based analysis. Exp Gerontol.

[30] Hu Z, Lin X, Chiwanda Kaminga A, Xu H (2020). Impact of the COVID-19 epidemic on lifestyle behaviors and their association with subjective well-being among the general population in Mainland China: Cross-sectional study. J Med Internet Res.

[31] Parry S, Straker L (2013). The contribution of office work to sedentary behaviour associated risk. BMC Public Health.

[32] Waters CN, Ling EP, Chu AHY, Ng SHX, Chia A, Lim YW, Müller-Riemenschneider F (2016). Assessing and understanding sedentary behaviour in office-based working adults: A mixed-method approach. BMC Public Health.

[33] (2020). Prevention Measures Against Coronavirus Disease 2019 (COVID-19).

[34] Zieff G, Bates L, Kerr Z, Moore J, Hanson E, Battaglini C, Stoner L (2020). Targeting sedentary behavior as a feasible health strategy during COVID-19. Transl Behav Med.

[35] Chtourou H, Trabelsi K, H'mida C, Boukhris O, Glenn JM, Brach M, Bentlage E, Bott N, Shephard RJ, Ammar A, Bragazzi NL (2020). Staying physically active during the quarantine and self-isolation period for controlling and mitigating the COVID-19 pandemic: A systematic overview of the literature. Front Psychol.

[36] Salari N, Hosseinian-Far A, Jalali R, Vaisi-Raygani A, Rasoulpoor S, Mohammadi M, Rasoulpoor S, Khaledi-Paveh B (2020). Prevalence of stress, anxiety, depression among the general population during the COVID-19 pandemic: A systematic review and meta-analysis. Global Health.

[37] Ettman CK, Abdalla SM, Cohen GH, Sampson L, Vivier PM, Galea S (2020). Prevalence of depression symptoms in US adults before and during the COVID-19 pandemic. JAMA Netw Open.

[38] De Boni RB, Balanzá-Martínez V, Mota JC, Cardoso TDA, Ballester P, Atienza-Carbonell B, Bastos FI, Kapczinski F (2020). Depression, anxiety, and lifestyle among essential workers: A web survey from Brazil and Spain during the COVID-19 pandemic. J Med Internet Res.

[39] Hyland P, Shevlin M, McBride O, Murphy J, Karatzias T, Bentall RP, Martinez A, Vallières F (2020). Anxiety and depression in the Republic of Ireland during the COVID-19 pandemic. Acta Psychiatr Scand.

[40] Ainsworth B, Caspersen C, Matthews C, Mâsse LC, Baranowski T, Zhu W (2012). Recommendations to improve the accuracy of estimates of physical activity derived from self report. J Phys Act Health.

[41] Smirmaul B, Arena R (2020). The urgent need to sit less and move more during the COVID-19 pandemic. J Cardiopulm Rehabil Prev.

[42] Peçanha T, Goessler KF, Roschel H, Gualano B (2020). Social isolation during the COVID-19 pandemic can increase physical inactivity and the global burden of cardiovascular disease. Am J Physiol Heart Circ Physiol.

[43] Pinto AJ, Dunstan DW, Owen N, Bonfá E, Gualano B (2020). Combating physical inactivity during the COVID-19 pandemic. Nat Rev Rheumatol.

